# The HF-AF ENERGY Trial: Nicotinamide Riboside for the Treatment of Atrial Fibrillation in Heart Failure Patients

**DOI:** 10.1007/s10557-022-07382-4

**Published:** 2022-10-13

**Authors:** Lisa Pool, Paul Knops, Olivier C. Manintveld, Jasper J. Brugts, Dominic A. M. J. Theuns, Bianca J. J. M. Brundel, Natasja M. S. de Groot

**Affiliations:** 1 Department of Cardiology, Erasmus Medical Center, Rotterdam, The Netherlands; 2 Department of Physiology, Amsterdam Cardiovascular Sciences Heart Failure and Arrhythmia, Amsterdam UMC, Vrije Universiteit Amsterdam, Amsterdam, The Netherlands

**Keywords:** Atrial fibrillation, Energy metabolism, Heart failure, Mitochondrial function, Nicotinamide riboside, Therapy

## Abstract

**Background:**

The presence of atrial fibrillation (AF) in heart failure (HF) patients with reduced ejection fraction is common and associated with an increased risk of stroke, hospitalization and mortality. Recent research findings indicate that a reduction in nicotinamide adenine dinucleotide (NAD^+^) levels results in mitochondrial dysfunction, DNA damage and consequently cardiomyocyte impairment in experimental and clinical HF and AF. The HF-AF ENERGY trial aims to investigate the cardioprotective effects of the NAD^+^ precursor nicotinamide riboside (NR) treatment in ischemic heart disease patients diagnosed with AF.

**Study design:**

The HF-AF ENERGY trial is a prospective intervention study. The study consists of a (retrospective) 4 months observation period and a 4 months intervention period. The cardioprotective effect of NR on AF burden is investigated by remote monitoring software of implantable cardiac defibrillators (ICDs), which enables continuous atrial rhythm monitoring detection. Cardiac dimension and function are examined by echocardiography. Laboratory blood analysis is performed to determine mitochondrial function markers and energy metabolism. All the study parameters are assessed at two fixed time points (pre- and post-treatment). Pre- and post-treatment outcomes are compared to determine the effects of NR treatment on AF burden, mitochondrial function markers and energy metabolism.

**Conclusion:**

The HF-AF ENERGY trial investigates the cardioprotective effects of NR on AF burden and whether NR normalizes blood-based mitochondrial function markers and energy metabolites of the NAD metabolome in ischemic heart disease patients diagnosed with AF. The study outcomes elucidate whether NAD^+^ metabolism can be used as a future therapy for HF patients with AF.

## Introduction

Atrial fibrillation (AF) in patients with heart failure (HF) due to reduced ejection fraction is commonly encountered and associated with an increased risk of stroke, hospitalization and all-cause mortality [1]. The global prevalence of AF is around 59.7 million (30.3 million males and 29.4 million females) [2], whereas HF affects around 64 million people worldwide [3]. The prevalence of AF in HF patients with reduced ejection fraction (HFrEF) ranges from 13 to 27% [4] and is associated with common risk factors, including advanced age, hypertension, diabetes, obesity and ischemic heart disease [4]. Molecular changes and consequently structural damage and impaired intra-atrial conduction (i.e. electropathology) predisposes the heart to both AF and HF [5]. However, the management of AF in HFrEF patients remains a therapeutic challenge.

Nicotinamide adenine dinucleotide (NAD^+^) has emerged as a new avenue for the development of metabolic therapy in HF patients [6]. NAD^+^ is important for mitochondrial adenosine triphosphate (ATP) generation and is the precursor for NADPH, which is required for reactive oxygen species (ROS) detoxification [7, 8]. In addition, NAD^+^ is an essential substrate for poly(ADP-ribose) polymerases (PARPs) [9]. PARP1 is activated in response to ROS-induced DNA lesions and as a consequence leads to depletion of myocardial NAD^+^ levels [7, 10]. In case of HF as well as AF, PARP1-induced NAD^+^ depletion is followed by mitochondrial and cardiomyocyte dysfunction [9, 11, 12]. Therefore, compounds that maintain NAD^+^ levels may represent an attractive therapeutic approach in disorders where increased PARP1 activity causes impaired cardiomyocyte and mitochondrial function and ultimately myocardial failure.

A recent study showed that supplementation with the NAD^+^ precursor nicotinamide riboside (NR) normalizes the NAD^+^/NADH ratio in healthy volunteers and has the potential as a therapy in patients with impaired cardiomyocyte and mitochondrial function [13]. Previous research already showed that NR protects against cardiomyopathy in patients with HF [6] and contractile dysfunction in experimental AF model systems [8, 9, 12, 14]. However, the therapeutic benefits of NR treatment have only been investigated in HF patients and experimental AF models and not yet in a clinical trial in HF patients with AF.

Therefore, the HF-AF ENERGY trial is the first clinical study that combines HF and AF patients. The HF-AF ENERGY trial aims to investigate whether NR normalizes blood-based mitochondrial function markers and energy metabolism and the cardioprotective effects of the NAD^+^ precursor NR (Tru Niagen®, ChromaDex) on AF burden in ischemic heart disease patients diagnosed with AF. Positive outcomes from this therapeutic study may represent a major breakthrough in the therapy of AF in patients with ischemic determined HF.

## Study Design

The HF-AF ENERGY study is a prospective interventional trial, with a scheduled duration of 36 months. The study is carried out in adherence to the declaration of Helsinki and is approved by the local medical ethical committee of the Erasmus Medical Center (MEC-2021–0734).

### Study Objectives

The aim of the study is to investigate whether HFrEF patients with paroxysmal or persistent AF benefit from NR treatment. The primary study objective is to investigate whether NR normalizes blood-based mitochondrial function markers and energy metabolites of the NAD metabolome, including NAD^+^/NADH. Our secondary objective is to examine whether NR reduces the burden of AF episodes in patients with ischemic heart disease.

### Study Population

For the HF-AF ENERGY study, 20 patients aged between 18 and 80 years with ischemic cardiomyopathy, diagnosed with paroxysmal or persistent AF, and an implantable cardiac defibrillator (ICD) equipped with continuous atrial rhythm registration are included. Patients are recruited at the outpatient clinic of Cardiology at the Erasmus Medical Center, Rotterdam, the Netherlands. Prior to enrolling, patients are provided with a written and oral explanation of the study procedure. Written consent is obtained from all patients.

### Inclusion Criteria

Patients need to meet the following inclusion criteria to be eligible to participate in this study: (1) aged between 18 and 80 years, (2) suffer from ischemic cardiomyopathy with left ventricular ejection fraction ≤ 40%, (3) diagnosed with paroxysmal or persistent AF, (4) received an ICD equipped with an atrial lead and remote rhythm monitoring and (5) no cardiac-related hospital admission within the 3 months prior to inclusion.

### Exclusion Criteria

Patients diagnosed with permanent AF, left ventricular ejection fraction (LVEF) > 40%, hemodynamic instability and malabsorption, metabolic or inflammatory diseases are excluded from participation in the HF-AF ENERGY trial. Patients scheduled for a cardiac intervention are excluded from participation. Moreover, patients with alterations in medical treatments during the intervention period, a left ventricular assist device, on the waiting list for a heart transplantation or history of intolerance to NR precursors, including niacin or nicotinamide, are also excluded from participation.

### Study Design

**Fig. 1:**
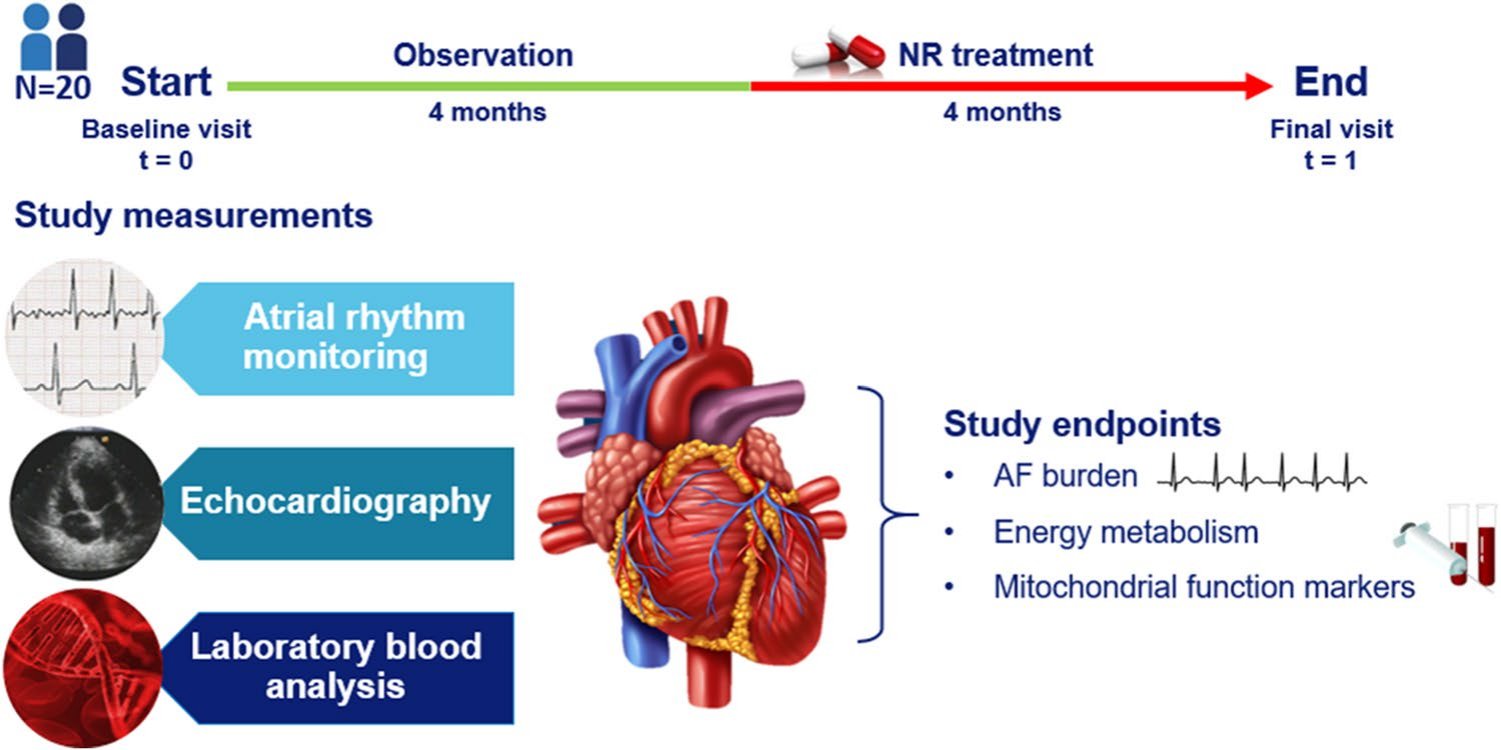
Clinical trial design. At baseline (*t* = 0), the average AF burden is calculated by remote rhythm monitoring using the atrial lead. Cardiac function is examined by echocardiography; energy metabolism and mitochondrial function marker are determined by laboratory blood analysis. After baseline, 4 months of NR supplementation follows. At follow-up (*t* = 1), the same clinical measurements are performed. Pre- and post-treatment outcomes are compared to determine the cardioprotective effects of NR on AF burden, mitochondrial function markers and energy metabolism

Twenty patients are enrolled in the HF-AF ENERGY trial to investigate the effect of NR (1000 mg, twice daily) treatment on AF burden, blood-based mitochondrial function markers and energy metabolism. The study consists of a 4 months observation period and a 4 months intervention period. At baseline, AF burden is determined for each patient by utilizing continuous atrial rhythm monitoring using the atrial lead. The ICD provides the exact time in AF, including hours, minutes and seconds. AF burden is calculated by the amount of time spent in AF divided by the total amount of time recorded for the observation period. Baseline levels of energy metabolites and mitochondrial function markers are derived from blood samples. Levels include NAD^+^/NADH [9] of the NAD metabolome, circulating mitochondrial DNA [15] and the oxidative DNA damage markers such as 8-hydroxy-2’-deoxyguanosine (8-OHdG) [16]. Echocardiographic examination is be performed to assess cardiac strain, systolic and diastolic function, including left atrial volume, E/A ratio, isovolumic relaxation time, right ventricular systolic pressure, LVEF and left ventricular volume/mass. Patient’s characteristics (e.g. age, medical history, medication and cardiovascular risk factors) are obtained from the patient’s medical file. After the baseline visit, patients start taking NR supplements for a period of 4 months. The supplements are supplied as 250 mg/capsule, to be administered orally. The initial dose is one capsule twice daily (500 mg), followed by weekly up-titration by one capsule/dose to a maximal dose of 4 capsules twice daily (2000 mg daily). At week 4, patients have reached the maximum doses, which are continued till final follow-up visit in week 16. At the final visit in week 16, patients undergo the same assessments as performed at the baseline visit. For all the study endpoints, pre- and post-treatment outcomes are compared to determine the effect of NR on AF burden, blood-based mitochondrial function marker and energy metabolism in patients with ischemic cardiomyopathy. The study flow chart is depicted in Fig. 1.

### Sample Size Calculation

So far, the AF burden and blood-based NAD^+^ levels have not been quantified in HFrEF patients with AF. Therefore, the sample size calculation is based on the NAD^+^ levels derived from blood samples of healthy volunteers [13]. We assume a likelihood of at least a 35% increase in NAD^+^ metabolite concentration at the final visit [13]. To compute the required sample size, the mean baseline NAD^+^ level and standard deviation (SD) are calculated for HF patients, derived from blood samples. With a type I error of 0.05 (two-sided), a power of 90%, a Pearson correlation coefficient of 0.5 and a bivariate normal distribution of the pre-post measurements, the initial required number of patients is 15. To account for dropout and uncertainties within the sample size calculation, the sample size is adjusted to 20 subjects for the HF-AF ENERGY trial.

## Main study Parameters

### Laboratory Blood Analysis

The primary study endpoint is the mitochondrial function markers and energy metabolites. Blood samples are collected pre- and post-NR treatment to determine blood-based energy metabolites of the NAD metabolome (e.g. NAD^+^/NADH) and markers of mitochondrial function, including oxidative DNA damage and circulating mitochondrial DNA. Energy metabolites are identified using liquid chromatography mass spectrometry (LC–MS) [17]. Circulating mitochondrial DNA gene expression is measured using the quantitative polymerase chain reaction method, as previously described [15], and oxidative DNA damage is detected by enzyme-linked immunosorbent assay (ELISA) [16]. The outcomes determine whether NR treatment normalizes energy metabolite levels and mitochondrial function markers in ischemic cardiomyopathy patients with AF.

### AF Burden

The burden of AF episodes is monitored using remote monitoring. For the purpose of the study, patients implanted with an ICD are enrolled as this manufactures has remote monitoring with daily transmission of data. The ICD with an atrial lead or atrial sensing electrode enables continuous atrial rhythm monitoring in order to calculate AF burden. Overall AF burden for the two separate study periods (pre- and post-treatment) is calculated to examine whether NR reduces the burden of AF episodes.

### Echocardiographic Examination

Echocardiographic assessment is performed to examine the effect of NR on cardiac dimension and function. For this purpose, cardiac strain, left atrial volume, E/A ratio, isovolumic relaxation time, right ventricular systolic pressure, LVEF, left ventricular size and volume and left ventricular diastolic function are measured.

## Study Endpoints

All the study parameters are assessed at two fixed time points: at the start of the intervention period (pre-treatment) and 4 months from the baseline examination time point (post-treatment). The primary endpoint is changes in blood-based energy metabolite concentrations (NAD^+^/NADH) and mitochondrial function markers. The secondary endpoint is the overall changes in AF burden during the 4-month treatment period. Based on the results of the primary and secondary endpoints, the cardioprotective effects of NR on energy metabolism, mitochondrial function markers and AF burden can be determined. These findings indicate whether the NAD^+^ precursor NR represents a novel therapeutic to prevent AF progression and improve cardiac function in ischemic heart disease patients by increasing blood-based mitochondrial markers and energy metabolite levels.

The primary hypothesis is that mitochondrial function markers and blood-based energy metabolites of the NAD metabolome (e.g. NAD^+^/NADH) are normalized after NR treatment. Secondarily, the AF burden decreases in HFrEF patients after NR treatment.

## Data Management

### Data Collection

Clinical data is collected and stored on a secured drive. The daily transmitted data by remote monitoring is downloaded from the password secured remote monitoring site of the manufacturer. Data is maintained in storage for a period of 15 years after completion of the study. The participants can withdraw from the study at any time without any consequences. The obtained data is used for the study, as per detail in the informed consent form.

### Confidentially

Collected data is secured against unauthorized access. A unique study code is assigned to each participant. The study is conducted in compliance with the guidelines of Good Clinical Practice, in full conformance with the Declaration of Helsinki and the law on Medical Research involving Human subjects.

## Statistical Analyses

Normally distributed continuous variables are expressed as mean ± SD, skewed continuous variables are expressed as median (minimum–maximum), and categorical variables are expressed as numbers (percentage). Normality is tested by the Shapiro–Wilk test. Paired *t* test or Wilcoxon one-sample test is used to compare continuous variable differences between pre- and post-treatment outcomes, depending on the normality of the distribution. A Poisson regression is used to study the effect of NR treatment on the number of AF episodes. All statistical analysis is performed at a significance level of 0.05.

## Discussion

The prevalence of AF in patients with HF is associated with an increased risk of ischemic stroke, hospitalization and mortality [18]. Given the high prevalence of AF in patients with HF, there is an unmet need for therapies that will reduce AF through novel mechanisms. Preliminary experimental studies suggest that the dietary supplement, NR, attenuates oxidative mitochondrial DNA damage and consequently improves cardiac function in HF and AF by normalizing blood-based mitochondrial function markers and energy metabolites of the NAD metabolome (e.g. NAD^+^/NADH) [9, 19–21]. These observations are consistent with the first clinical trial, demonstrating the beneficial effects of increased NAD^+^ blood levels on inflammation and potentially myocardial mitochondrial function in HF patients [6]. The HF-AF ENERGY trial is the first clinical trial that involves patients who have both HF and AF to examine the cardioprotective impact of NR treatment on AF burden and blood-based mitochondrial function markers and energy metabolism, and as such, this study addresses a gap in current knowledge on the clinical proof of concept for the NAD pathway as a druggable target in AF.

## Conclusion

Targeting energy metabolism by increasing NAD^+^ levels with NR could be a promising therapy for HF patients with AF. The HF-AF ENERGY trial investigates whether NR treatment attenuates AF burden and improves mitochondrial function markers and energy metabolism in ischemic cardiomyopathy patients with a reduced ejection fraction and paroxysmal or persistent AF. The study outcomes elucidate whether the energy metabolism can be used as a future therapy for ischemic determined HF patients with AF.

## Data Availability

The data underlying this article will be shared on reasonable request to the corresponding author.
